# Function After Surgical Treatment of Calcaneal Fractures Using Various Methods

**DOI:** 10.3390/jcm15093410

**Published:** 2026-04-29

**Authors:** Igor Kowal, Łukasz Tomczyk, Andrzej Bobiński, Krystian Kazubski, Piotr Morasiewicz

**Affiliations:** 1 Department of Orthopaedic and Trauma Surgery, Multidisciplinary Hospital in Zgorzelec, Lubańska 11-12, 59-900 Zgorzelec, Poland; 2 Department of Food Safety and Quality Management, Poznan University of Life Sciences, Wojska Polskiego 31, 60-624 Poznan, Poland; 3 Department of Orthopaedic and Trauma Surgery, Institute of Medical Sciences, University of Opole, al. Witosa 26, 45-401 Opole, Poland

**Keywords:** calcaneal fracture, Ilizarov method, plate fixation, range of motion, activity scale

## Abstract

**Background:** The impact that various methods of calcaneal fracture treatment have on functional outcomes is not thoroughly understood. The purpose of this study was to analyze the impact of calcaneal fractures on the level of physical activity and foot and ankle mobility following various fixation methods. **Methods:** In our two-center retrospective analysis, we compared treatment outcomes of intra-articular calcaneal fractures in 50 patients treated with the Ilizarov method and 49 patients who underwent internal plate fixation. The following parameters were analyzed: range of motion of the foot and ankle joint; the UCLA Activity Scale, the Saltin–Grimby scale, and the Visual Analog Scale (VAS) of Activity. No multivariable adjustment was performed. Accordingly, between-group comparisons are unadjusted, except where stratified analyses are indicated. **Results:** Postoperatively, a significant improvement was noted in terms of UCLA Activity parameters and the Saltin–Grimby score in the Ilizarov group. A comparison of post-treatment physical activity levels in the two groups revealed significantly better scores in the Grimby scale and VAS activity in the Ilizarov group than in the internal fixation group. The ranges of motion in the Ilizarov group showed significantly worse mobility in the operated limb than in the intact limb. The ranges of dorsiflexion, foot inversion, and foot eversion in the ORIF group were comparable for the treated and intact limb. A comparison of ranges of motion in the study groups showed significantly lower ranges of motion in the Ilizarov group than in the ORIF group. **Conclusions:** Calcaneal fracture treatment with the Ilizarov method may be associated with better physical activity levels than internal plate fixation. Foot and ankle mobility following calcaneal fracture treatment is better in patients treated with internal plate fixation. The results should be taken with caution due to the lack of randomization, two-center design and the retrospective nature of the study.

## 1. Introduction

Calcaneal fractures, which considerably affect the function of the foot and ankle, are a serious orthopedic concern. These injuries account for about 2% of all bone fractures and represent 60–75% of tarsal fractures [1,2]. Due to the complex anatomy and biomechanics of the calcaneus, such injuries often result in long-term pain, limited range of motion, and impaired gait [3]. Calcaneal fractures are most commonly due to falls from height or traffic accidents, which generate strong axial forces acting on the foot and determine the fracture type [4].

As part of the hindfoot, the calcaneus plays a key role in load distribution, shock absorption, and initiation of the stance phase during walking [5]. Therefore, structural integrity of the calcaneus is necessary for normal foot and ankle joint biomechanics. Calcaneal fractures, particularly Sanders type III and IV fractures with marked bone fragment displacement, may alter subtalar joint geometry and the lower limb axis, and facilitate the development of osteoarthritis, which poses a major functional problem for the patient [6].

Calcaneal fracture treatment poses a challenge for orthopedic surgeons, with choice of the therapeutic method depending on multiple factors, such as fracture type, bone fragment displacement, soft tissue injury, and the general health of the patient. Both conservative and surgical treatment have their advantages and drawbacks, and the ultimate treatment outcome often depends on a precise reconstruction of calcaneal anatomy and early rehabilitation [3,7,8].

Direct comparisons focusing specifically on physical activity levels and ankle and foot mobility following various fixation methods in calcaneal fracture patients are limited in the literature. Apart from clinical and radiological assessments, functional assessment is also important for patients receiving post-traumatic musculoskeletal injury treatment, their orthopedic surgeons, and their rehabilitants [3,6,9]. There is no assessment of sports and physical activity of patients after treatment of calcaneal fractures. Only a few, mostly retrospective, single-center studies have directly compared open reduction and internal fixation (ORIF) with the Ilizarov method in the treatment of calcaneal fractures, typically including relatively small patient cohorts. Larger, well-designed comparative trials between these two strategies are lacking, which underscores the need for the present analysis. Previous studies are mostly retrospective, single-center, or subject to center-specific selection and rehabilitation practices, few have used prospective, randomized allocation or applied robust adjustment (e.g., multivariable regression or propensity-score methods) to control for baseline differences. We hypothesized that different fixation techniques for calcaneal fractures would influence functional outcomes. This study aimed to evaluate how various fixation methods affect physical activity levels and foot and ankle mobility after calcaneal fracture treatment.

## 2. Material and Methods

### 2.1. Study Design and Patient Selection

This study was a retrospective medical record review combined with prospective clinical follow-up. This two-center study spanned different time periods because of the low incidence of calcaneal fractures at each institution. Treatment allocation was center-based rather than randomized, and case accrual and timing depended on the surgically treated fractures available at each center. We evaluated the outcomes of treating intra-articular calcaneal fractures using plate osteosynthesis or a Polish modification of the Ilizarov method [9,10]. At one center, between 2019 and 2025, 60 intra-articular calcaneal fractures were treated with internal plate fixation (VariAx Calcaneal Plate, Stryker, Kalamazoo, MI, USA). At the other center, between 2021 and 2025, 59 such fractures were treated using the Polish modification of the Ilizarov external fixation [9,10].

Inclusion criteria for the study were Sanders type II, III, or IV fractures and an absence of prior fractures or concomitant disorders affecting the same lower limb. The follow-up lasted a minimum of two years after treatment completion, and inclusion required informed consent, complete medical and radiological records, and full functional and foot mobility assessment data. Exclusion criteria were prior fractures of the same foot or lower limb, lower limb comorbidities, follow-up shorter than two years, or absence of informed consent. Statistical data on preoperative height and weight were not available and are currently inaccessible; therefore, they were not included in the study. All participants were informed that participation was voluntary and that they could withdraw at any time. The study was approved by the local ethics committee and conducted in accordance with the Declaration of Helsinki.

Patients who had previously undergone treatment for intra-articular calcaneal fractures at two different centers were retrospectively reviewed. From one center, 49 patients (all males; mean age 48 years; range 23–68 years) treated with internal plate fixation were included. From the other center, 50 (1 female, 49 males; mean age 49 years; range 28–73 years) patients treated with the Polish modification of the Ilizarov external fixation were included, based on the defined inclusion and exclusion criteria. No statistically significant age and BMI differences were found between the groups. The distribution of Sanders fracture types in the internal fixation group was 12 type II, 25 type III, and 12 type IV. In the Ilizarov group, there were 12 type II, 26 type III, and 12 type IV Sanders fractures.

### 2.2. Surgical Procedures

Each internal plate fixation procedure was carried out in the lateral or prone position by one of two experienced orthopedic surgeons. An extended lateral approach was used, and neurovascular structures and fibular muscle tendons were protected with levers and a 1.6 mm Kirschner wire, used as a retractor. Subsequently, the articular surface was realigned and bone fragments were fixed with Kirschner wires and a plate secured with locking cortical screws, under intraoperative fluoroscopy.

Each Ilizarov treatment procedure was performed under spinal anesthesia in a supine position by the same orthopedic surgeon. The surgical technique, the structure of the Ilizarov fixator, and the course of treatment with a Polish modification of the Ilizarov method have been thoroughly described in earlier papers [9,10]. After the rings were affixed to the tibia and fibula, a Kirschner wire was inserted into the calcaneal tuberosity under intraoperative fluoroscopy. The wire was subsequently connected to the foot half-ring, which was attached to the distal leg ring. Finally, a closed reduction was conducted with fluoroscopic assistance. This adjusted spatial arrangement of the fixator enabled the realignment of fracture fragments in both the frontal and sagittal planes [9,10].

### 2.3. Postoperative Management

The treated limb of each patient who underwent internal plate fixation was initially stabilized in a leg cast until the first follow-up visit and suture removal on postoperative day 14. The patients ambulated with two elbow crutches. After the first radiological follow-up, the cast was replaced with a walker splint. The subsequent follow-up was scheduled for week 6 unless there were problems with surgical wound healing. After the cast was replaced with a walker splint, patients were permitted to gradually increase weight-bearing, initially constrained by manageable pain levels. Adjustments to management were made based on follow-up X-ray images, with the goal of achieving full weight-bearing on the affected limb by the third month postoperatively. Patients who underwent the Ilizarov procedure were able to start walking with elbow crutches and partial weight-bearing from the first day after surgery. As pain diminished, an incremental increase in weight-bearing was encouraged to enhance rehabilitation. Follow-up X-ray images were obtained every 4–6 weeks. The progress of wound healing was evaluated through both radiological and clinical observations, such as the absence of pain or abnormal mobility. Once there was radiological and clinical confirmation of bone union, the Ilizarov fixator was loosened to facilitate the transition to full weight-bearing on the affected limb. If clear radiological and clinical indications of bone union were present, the fixator was removed unless there was evidence of secondary displacement of bone fragments [9,10].

### 2.4. Outcome Measures

We analyzed the following foot and ankle parameters: dorsiflexion, plantar flexion, foot inversion, and foot eversion. The patients’ physical activity levels were assessed with the UCLA Activity Scale, Saltin–Grimby scale, and the Visual Analog Scale (VAS) of Activity AOFAS and VAS pain scale. Joint ranges of motion were not assessed systematically, and no single standardized measurement protocol was applied across the centers. Pre-injury physical activity level and exertional tolerance were not influenced or controlled by the investigators and instead reflect real-world variation among patients presenting with calcaneal fractures. Furthermore, we did not perform systematic ROM measurements or collect questionnaires and PROMs from healthy control subjects.

The Range of Motion (ROM) was measured with an electronic goniometer, with each parameter measured three times. The means of the three measurements were used for statistical analysis. The UCLA Activity Scale is a single-item 10-point scale used for assessing the level of physical activity, particularly in patients after lower limb surgery. A score of 10 indicates a high level of physical activity, and 1 indicates a very low level of physical activity, with the patient requiring aid from others and being unable to leave the house on their own [11]. The Saltin–Grimby scale, also known as the Saltin–Grimby Physical Activity Level Scale (SGPALS), is a tool for assessing the level of leisure-time physical activity. The scale classifies the patient’s self-reported level of physical activity into one of four categories: physically inactive, some light physical activity, regular physical activity and training, or regular hard physical training for competition sports [12]. The VAS measures a subjective level of activity, with the patient assessing their perceived level of activity on a linear 10 cm scale, where 0 means no physical activity, and 10 means very intense training. This last scale is simple and commonly used in medicine to assess treatment effectiveness [13]. Functional outcome was assessed using the AOFAS (American Orthopaedic Foot and Ankle Society) score (0–100 points; higher scores indicate better function). Pain intensity was measured with the Visual Analogue Scale (VAS) ranging from 0 to 10, where 0 denotes no pain and 10 the worst imaginable pain. AOFAS and VAS measurements were obtained at predefined time points (postoperative follow-ups). Results are presented as medians and interquartile ranges. We deliberately focused on objective measures of foot and ankle function that were routinely and consistently collected at both centers. Not all functional scores mentioned in the literature or by the reviewer (e.g., broader PROMs, comprehensive pain VAS) were available for this retrospective cohort. The selected scales and questionnaires were considered by the authors to be the most relevant for assessing foot function after calcaneal fracture treatment and were those that could be applied uniformly across both institutions. We used the AOFAS and available activity scales recorded in the medical records. We did not collect foot- and ankle-specific PROMs, such as FFI or FAOS; this limits direct comparability with studies using those instruments.

The gathered data were processed, stored, and utilized for statistical analysis. We compared the ranges of motion and physical activity levels between patients who underwent internal fixation and those who received Ilizarov external fixation. The term ‘before treatment’ in this manuscript refers to the state before the injury and prior to initiation of an operative intervention. Pre-injury data (AOFAS, ROM, VAS pain) were not collected. Activity levels labeled ‘before treatment’ were obtained at the post-injury visit via questionnaire/clinical interview and refer to the state before the injury and prior to initiation of an operative intervention.

### 2.5. Statistical Analysis

Statistical analyses were performed using Statistica version 14.1. Distribution normality was assessed with the Shapiro–Wilk test. Quantitative variables were compared using the independent-samples *t*-test or the Mann–Whitney U test, as appropriate. Statistical significance was defined as *p* < 0.05. All comparisons between groups are unadjusted unless stated otherwise. Continuous variables are reported as mean (SD) or median (IQR) and were compared using a Student’s *t*-test or Mann–Whitney U test as appropriate. No multivariable regression or propensity-score matching was performed due to the sample size and retrospective design constraints. To partially assess the influence of baseline differences, we performed predefined stratified analyses by key variables (Sanders classification II/III/IV, age categories, and presence of comorbidities) and sensitivity analyses excluding extreme outliers. These analyses are presented in the Results. Given the observed baseline differences in physical activity between groups, we considered the potential impact of regression on the mean, selection bias, and indication bias. Accordingly, we report on both baseline and postoperative values for activity-related variables. More extensive multivariable adjustment was limited by the sample size and heterogeneity of the cohort, and residual confounding cannot be excluded.

## 3. Results

**Figure 1:**
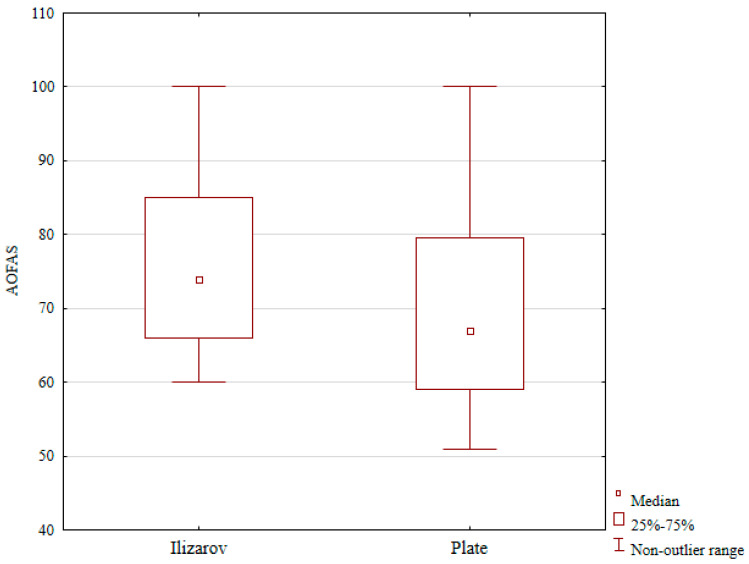
AOFAS scale in Ilizarov and ORIF group.

The median postoperative AOFAS score was 74 in the Ilizarov group versus 67 in the plate group (differences were statistically significant, *p* < 0.01); see Table 1 and Figure 1. The media postoperative VAS pain score was 2 in the Ilizarov group and 5.5 in the plate group; this difference was not statistical (see Table 1).

Physical activity assessment details from both evaluated groups have been presented in Table 2 and Table 3. Statistical analysis revealed a significant improvement in terms of UCLA Activity parameters (from a median preoperative score of 4 to a postoperative score of 6; *p* = 0.042) and the Saltin–Grimby score (from a median preoperative score of 3 to a postoperative score of 4; *p* = 0.001) in the Ilizarov group. We also observed an improvement in the VAS activity score, but the latter change was not statistically significant, Table 2 and Table 3.

**Figure 2:**
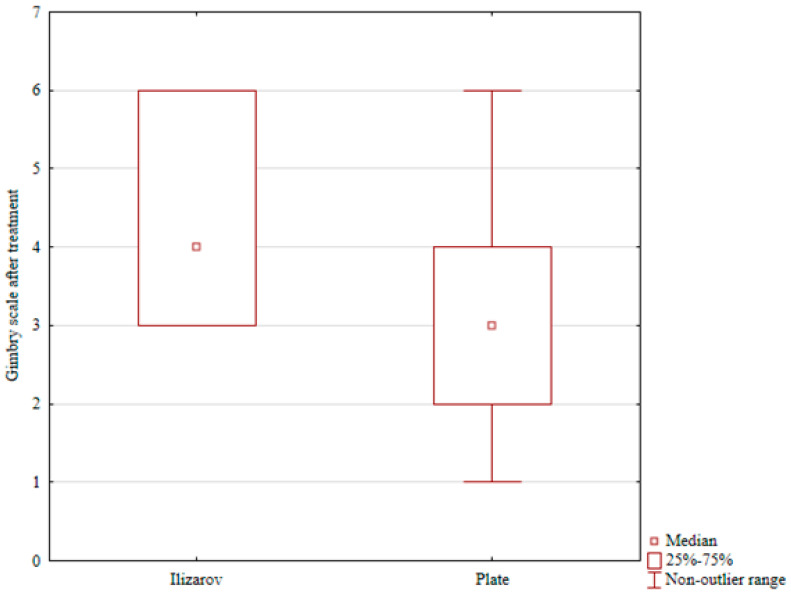
Grimby scale after treatment in the Ilizarov group and in the internal fixation group.

**Figure 3:**
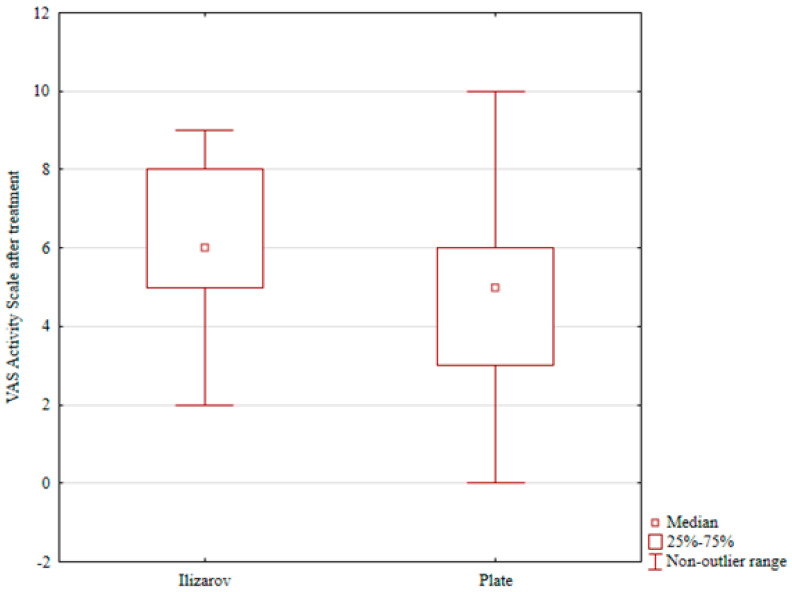
VAS-Activity after treatment in the Ilizarov group and in the internal fixation group.

Conversely, the patients treated with open reduction and internal fixation (ORIF) showed postoperative lowering of the scores in all three physical activity scales (UCLA, Grimby, and VAS activity), and the differences were statistically significant (*p* = 0.001, *p* = 0.001, and *p* = 0.001, respectively; see Table 2 and Table 3). A comparison of post-treatment physical activity levels in the two groups revealed significantly better scores in the Grimby scale and VAS activity in the Ilizarov group than in the internal fixation group (*p* = 0.001 and *p* = 0.03, respectively; see Table 4, Table 5 and Table 6 and Figure 2 and Figure 3).

A comprehensive comparison of the ranges of motion in both the treated and unaffected limbs in the two groups is presented in Table 4, Table 5 and Table 6 as well as in Table 7 and Table 8. Significant differences were observed in the ranges of dorsiflexion, plantar flexion, and foot inversion within the Ilizarov group between the treated and unaffected limb (medians of 18° vs. 40°, 15° vs. 30°, and 10° vs. 14°, respectively), with *p*-values of *p* < 0.001, *p* < 0.001, and *p* = 0.005, respectively. However, the range of foot eversion was found to be similar between the treated and unaffected feet, and these differences were not statistically significant, Table 3 and Table 4.

**Figure 4:**
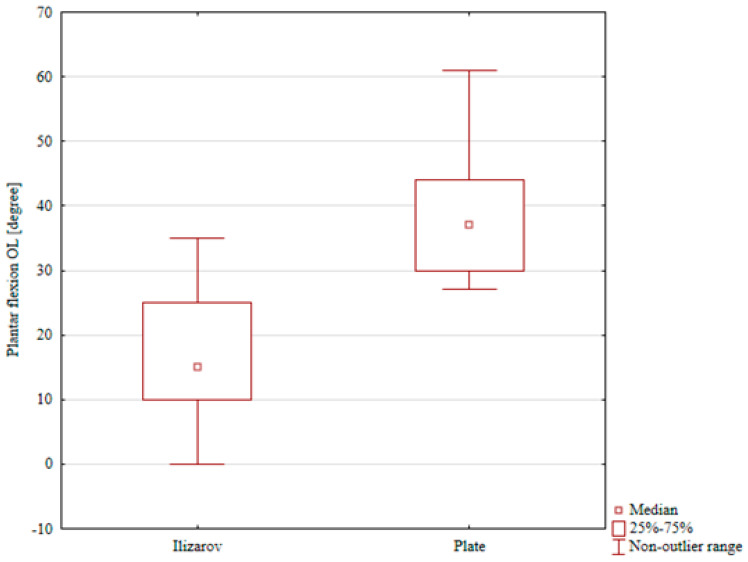
Plantar flexion in the Ilizarov group and in the internal fixation group.

**Figure 5:**
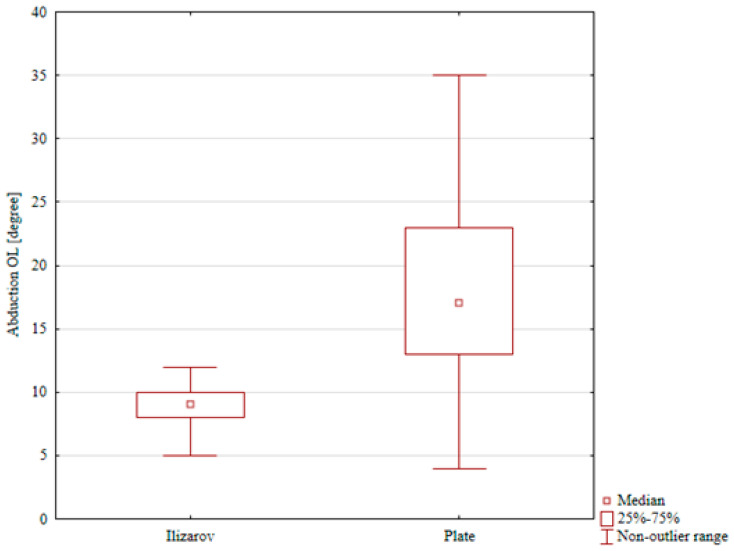
Foot eversion in the Ilizarov group and in the internal fixation group.

**Figure 6:**
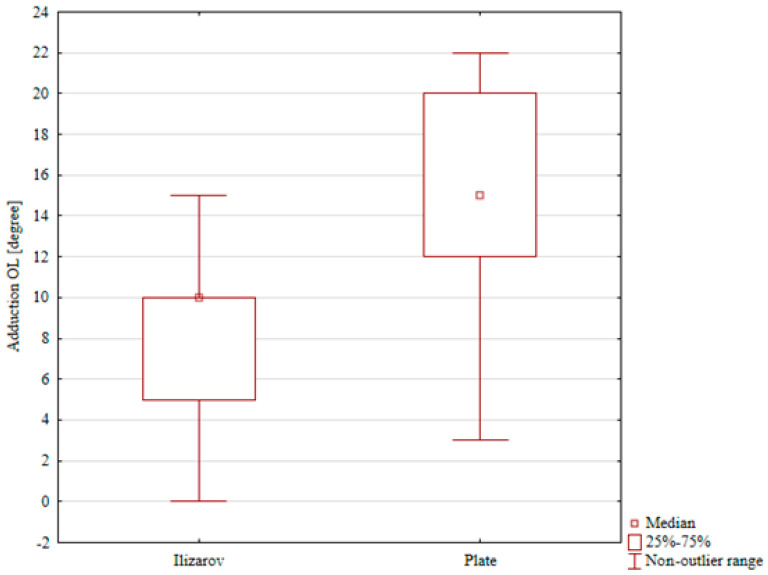
Foot inversion in the Ilizarov group and in the internal fixation group.

The ranges of dorsiflexion, foot inversion, and foot eversion in the internal fixation group were comparable for the treated and intact limb (a median of 13° vs. 16°, 15° vs. 17°, and 17° vs. 18°, respectively); these differences were not statistically significant. The only statistically significant result in the ORIF group was reduced plantar flexion in the treated limb (a median of 37°) in comparison with that in the intact limb (a median of 47°), *p* = 0.048), Table 4, Table 5, Table 6, Table 7 and Table 8. A comparison of ranges of motion in the study groups showed significantly lower ranges of plantar flexion, foot eversion, and foot inversion in the Ilizarov group than in the ORIF group (*p* < 0.001, *p* < 0.001, and *p* = 0.002, respectively), Table 4, Table 5, Table 6, Table 7 and Table 8, Figure 4, Figure 5 and Figure 6. Although several differences in ankle and foot ROM reached statistical significance, some of the absolute angular differences were small, in the order of only a few degrees. The clinical relevance of these small ROM deficits is therefore uncertain.

## 4. Discussion

This study aimed to evaluate and compare foot and ankle ranges of motion and self-reported physical activity levels in patients with calcaneal fractures treated with either the Ilizarov method or ORIF. Our statistical analysis revealed a significant postoperative improvement in UCLA and Grimby activity scores within the Ilizarov group, with VAS activity also improving but not reaching statistical significance. Conversely, the results for the ORIF group demonstrated a significant postoperative decline in all three physical activity scales (UCLA, Grimby, and VAS activity) compared to their preoperative baseline. A direct comparison of post-treatment physical activity levels between the two groups showed significantly better scores for both Grimby and VAS activity in the Ilizarov group compared to the ORIF group. Regarding ranges of motion, comparisons indicated that Ilizarov-treated patients exhibited considerably lower dorsiflexion, plantar flexion, and foot inversion in the operated limb compared to the intact limb, while foot eversion was comparable. For the ORIF group, dorsiflexion, foot inversion, and foot eversion were largely comparable between the treated and intact limbs, with only plantar flexion showing a significant reduction in the treated limb. Critically, an adjusted inter-group comparison of range of motion revealed significantly lower foot plantar flexion, foot eversion, and foot inversion in the Ilizarov group compared to the ORIF group.

In the analyzed cohort of patients treated for calcaneal fractures, the Ilizarov method was associated with better AOFAS functional outcomes. These divergent trends should be interpreted with caution. The postoperative decline in activity in the ORIF group and improvement in the Ilizarov group may partly reflect regression toward the mean, center-based treatment allocation, and indication bias rather than a pure effect of the fixation method itself. In addition, patients treated with the Ilizarov method may have had different expectations, more severe soft-tissue compromise, or distinct rehabilitation patterns, all of which could influence both activity scores and range-of-motion outcomes. 

Previous studies [3,6,9,14,15,16,17,18,19,20] have consistently reported that calcaneal fracture management can lead to reductions in joint range of motion and overall physical activity. However, direct comparisons of post-treatment activity levels and foot and ankle mobility across different fixation techniques, particularly with robust baseline adjustments, have been limited. Our findings contribute to addressing this gap by providing an adjusted comparative analysis of functional outcomes, highlighting distinct advantages and limitations for both the Ilizarov and ORIF methods. Effective rehabilitation, including early initiation of mobility exercises, muscle strengthening, and proprioception training, is well recognized as playing a key role in improving foot and ankle function, reducing pain, and accelerating the return to physical activity [21,22,23,24,25]. The observed differences in activity levels and range of motion in our study emphasize the complex interplay between surgical technique and the critical importance of a tailored rehabilitation approach in achieving optimal functional recovery.

In the context of varied outcomes and the complexities inherent in calcaneal fracture management, our study contributes a direct comparison of two distinct fixation philosophies. Biz et al. [14], in their retrospective analysis, compared outcomes of ORIF with percutaneous screw fixation for intra-articular calcaneal fractures, finding better radiological and functional outcomes for ORIF. They underscored the importance of patient-specific risk factors and noted a lower complication risk for ORIF in their cohort, devoid of patients with diabetes or open fractures. While Biz et al. [14] observed superior functional results with ORIF, our adjusted findings present a more nuanced picture: patients treated with ORIF showed a postoperative lowering of activity scores across all three scales (UCLA, Grimby, VAS activity compared to their preoperative baseline. This contrasts with Biz’s more broadly favorable ORIF outcomes and suggests that while anatomical restoration is a goal, the functional recovery, particularly in terms of activity levels, may be more variable and impacted by initial patient characteristics. This highlights the critical need for baseline adjustments, which we have performed, to interpret such comparisons accurately. The literature also showcases highly specialized interventions for complex cases [25,26,27]. Heinig et al. detailed a case report of a Sanders type IV calcaneal fracture managed with an external circular fixator alongside an antibiotic cement spacer, bioactive glass, and calcaneal osteotomy [25]. This multifaceted approach was designed to minimize vascular injury, prevent infection, and facilitate wound closure, ultimately leading to complete recovery in five months. Our study, by examining the Ilizarov method, a form of external fixation, within a broader patient cohort, provides a comparative perspective on similar principles of minimizing tissue disruption. Our adjusted results indicate that the Ilizarov group achieved significantly better post-treatment physical activity levels (Grimby and VAS Activity scores) compared to the ORIF group, aligning with the concept that less invasive approaches can support functional recovery. However, this must be balanced with the finding that the Ilizarov group demonstrated significantly worse ranges of motion in the operated limb compared to the intact limb and, critically, lower ranges of motion in plantar flexion, foot eversion, and foot inversion compared to ORIF group. This suggests that while external fixation may support activity, it might compromise joint mobility, a trade-off that highly complex cases like Heinig’s manage with tailored approaches. Our previous work, Pelc et al. [9], focused on evaluating functional outcomes of the Polish modification of the Ilizarov technique in 21 patients. That study reported significant post-treatment improvements in physical activity (UCLA, Grimby, and FFI-R scores) but noted persistent reductions in dorsiflexion, plantarflexion, and inversion. The current study extends these previous observations by providing a direct comparative analysis with ORIF, utilizing adjusted baseline characteristics. Our adjusted findings are largely consistent with our prior work for the Ilizarov group, showing improved activity scores, but importantly they now explicitly demonstrate a significantly better post-treatment activity profile for the Ilizarov method compared to ORIF. This robust comparison, addressing critical baseline differences, reinforces the efficacy of the Polish Ilizarov modification in promoting physical activity while concurrently confirming its association with specific range-of-motion deficits. Further supporting the efficacy of calcaneal fracture treatments, other studies [9,28,29,30,31,32,33] have also concluded that there are improved functional outcomes. These findings, along with our results, collectively emphasize the role of treatments like the Ilizarov method in enhancing foot function and physical activity. Park et al. and many other authors [9,21,28,34,35] highlight the importance of early rehabilitation, and our current data strengthen this, showing that despite improvements in foot function and physical activity with the Ilizarov method, persistent ankle motion deficits remain, indicating a clear need for more intensive, individualized rehabilitation strategies. This suggests that while implant burden can be reduced, comprehensive postoperative management remains paramount. 

Arsovski et al. [35], through case reports on ORIF-treated intra-articular calcaneal fractures, explored the relationship between anatomical restoration (e.g., Böhler’s angle) and functional outcomes (AOFAS scale). They reported favorable functional results linked to precise anatomical restoration and stressed the importance of meticulous surgical technique and proper patient selection. While other authors found favorable functional outcomes with ORIF [35,36], our analysis reveals a different aspect: the ORIF group in our study experienced a significant lowering of post-treatment activity scores compared to their own preoperative baseline. However, this group maintained better overall range of motion compared to the Ilizarov group. This discrepancy suggests that while anatomical restoration via ORIF may preserve range of motion to a greater extent, it does not automatically translate into superior activity levels, particularly if patient selection (as highlighted by Arsovski et al.) or specific rehabilitation protocols differ. Lastly, long-term functional outcomes after surgically treated intra-articular calcaneal fractures have been studied by Potter et al. [37] in a large cohort (*n* = 73) with a mean follow-up of 12.8 years. They assessed various scales, including AOFAS and FFI (foot- and ankle-specific PROMs which were not included in our current study, representing a limitation), no worse outcomes for high-energy trauma (traffic accidents) and a significant proportion (18%) requiring additional procedures. Potter et al. also briefly mentioned a study by Buckley et al. [3] which found ORIF to be superior to conservative treatment but only in patients not receiving workers’ compensation. Our current study, with its adjusted mid-term data, offers a relevant contribution by showing no significant correlation between treatment outcomes and workers’ compensation status in our cohort, aligning with the more nuanced view presented by Buckley et al. [3]. Our comparative, findings provide specific insights into activity and range-of-motion differences at a mid-term follow-up, complementing the long-term perspective of Potter et al. and further informing clinical decision-making regarding the functional trade-offs of each method.

Our findings indicate that treatment with the Ilizarov method leads to improved post-treatment physical activity. Patients treated with the Ilizarov technique achieved higher activity scores than those who underwent ORIF. This benefit may be due to earlier weight-bearing permitted by the Ilizarov construct and the avoidance of scarring and adhesions associated with open surgery. Conversely, apprehension about loading the injured limb may have restricted activity in the ORIF group. We found reduced foot and ankle ranges of motion in Ilizarov-treated patients compared with ORIF-treated patients. Comparison between the operated and contralateral limbs also showed poorer mobility outcomes in the Ilizarov group. These limitations may result from the design of the Ilizarov frame and aspects of the surgical technique that constrain joint motion during treatment, as well as from less precise anatomical restoration of fracture fragments (the goal being an approximate calcaneal shape). Overall, our results support the need for more intensive and prolonged rehabilitation after calcaneal fracture, regardless of fixation method.

Calcaneal fractures constitute a major medical and social problem [20,22,23,38,39]. A comprehensive treatment approach, involving accurate diagnostics, personalized selection of the treatment method, and intensive rehabilitation, is essential for improving functional outcomes and minimizing the negative impact of calcaneal fractures on patients’ quality of life and social functioning.

Further studies are needed to develop more effective methods of treatment and rehabilitation for patients with calcaneal fractures. Such studies would help patients resume their occupational and other physical activities sooner and improve their socioeconomic status.

Our study is limited by its retrospective, non-randomized, center-based design. Treatments were allocated by center and period, introducing a high risk of selection bias, center-related bias, and confounding. We did not perform multivariable adjustment (e.g., multivariable regression or propensity-score methods) because of dataset and sample size limitations; consequently, observed differences cannot be causally attributed to surgical technique. Differences in baseline patient characteristics, surgical indications, timing of surgery, and postoperative rehabilitation protocols between centers may largely explain the divergent activity outcomes (ORIF decrease vs. Ilizarov improvement). We also lacked commonly used validated foot- and ankle-specific PROMs (e.g., FFI, FAOS), limiting comparability with other series. These limitations should be considered when interpreting our findings. Due to the very rare occurrence of calcaneal fractures and the different practice locations, we decided to compare the treatment outcomes of calcaneal fractures between two centers. Only patients with Sanders type II, III and IV fractures underwent surgery; we had no influence on the patients’ characteristics—nor does anyone—and the rehabilitation protocols were tailored appropriately to the treatment method and its characteristics. Similar or smaller cohorts have been reported in other studies of calcaneal fracture outcomes [9,10,14,15,23,25,36]. Limitations related to the measurement instruments should be acknowledged. The AOFAS score includes investigation-rated components, which may introduce subjectivity and inter-observer variability; a lack of full standardization across centers may limit comparability. The VAS, while sensitive to changes in pain intensity, does not capture functional status or quality of life and is susceptible to timing of assessment and analgesic use. Future studies should consider standardized assessor training and the inclusion of patient-reported quality-of-life measures. The retrospective design and absence of pre-injury functional baseline data represent another limitation; baseline measurements were not feasible given the sudden occurrence of these injuries. The limitations of this study should be emphasized. First, pre-injury data were not collected; ‘before treatment’ refers to the state before the injury and prior to initiation of an operative intervention. We evaluated trauma patients who had sustained calcaneal fractures. Therefore, it was not possible to predict which patients would sustain a calcaneal fracture and evaluate them prospectively (ROM, VAS pain scale, other scales) before the injury. Other publications evaluating calcaneal fractures also evaluate patients only retrospectively [9,10,14,15,23,24,25,27,28,30,32,33,34,37,39], as it is impossible to predict which patients will sustain a fracture. Second, treatment allocation was center-based (different centers/time windows) rather than randomized, further increasing the risk of selection bias. Since treatment allocation depended on the treating center and was not randomized, there is a potential influence of selection bias, center-specific practices, and rehabilitation differences. These factors may have contributed to the observed differences between groups. Therefore, between-group comparisons should be interpreted with caution. Future prospective randomized or multicenter studies with harmonized inclusion criteria and collection of pre-injury data are recommended. We emphasize that this study did not include foot-/ankle-specific patient reported outcome measures (PROMs), such as FFI or FAOS. The absence of these instruments limits interpretation of claimed functional benefits and comparability with other studies. We recommend inclusion of these PROMs and complementary functional tests in future research. Treatment allocation was center-based and not randomized. Different centers may have applied different selection criteria and rehabilitation policies, potentially influencing patient selection (e.g., more severe soft tissue compromise) and outcomes. This is an important limitation and underscores the need for randomized or harmonized multicenter trials.

Strengths of the study include a direct comparison of two surgical approaches for calcaneal fractures and a two-center design with comparable patient populations in terms of size, mean age, follow-up duration, and sex distribution. Both centers applied similar rehabilitation protocols and follow-up schedules, and all surgeries were performed by only three orthopedic surgeons (two responsible for internal plate fixation and one for Ilizarov procedures), reducing inter-operator variability. For future work, we plan to expand the sample size and include gait analysis in subsequent studies.

## 5. Conclusions

Treatment of calcaneal fractures using the Ilizarov technique may be associated with superior post-treatment physical activity compared with internal plate fixation. Conversely, patients who underwent internal plate fixation demonstrated better foot and ankle mobility than those treated with the Ilizarov method. Regardless of fixation type, rehabilitation should be more prolonged and intensive after calcaneal fracture. Future prospective, multicenter studies with randomized or stratified allocation, standardized ROM assessment, and comprehensive collection of PROMs and pain VAS, ideally including healthy control reference data, are needed to determine whether the possible differences truly represent clinically meaningful functional advantages of one treatment protocol over the other.

## Figures and Tables

**Table 1:** Selected clinical and radiological parameters of patients with plate stabilization and patients using the Ilizarov method.

*p*	Z	Ilizarov Group (*n* = 50)	Plate Group (*n* = 49)		Analyzed Variable
0.167	1.381	0	3.5	Q1	VAS pain scale
0.241	1.171	2	5.5	Median	
0.002	−3.033	4	7.5	Q3	
*p* < 0.001	5.113	66	59	Q1	AOFAS
0.167	−2.920	74	67	Median	
0.241	1.381	85	79.5	Q3	

Z—standardized value of the Mann–Whitney test; *p*-value for the Mann–Whitney U test or Pearson ch2; Q1, Q3—1st and 3rd quartile.

**Table 2:** Detailed functional assessment of patients before and after surgery—Ilizarov group.

*p* Value	Z	After Treatment	Before Treatment		Analyzed Variable
Ilizarov Group					
0.042	−2.032	5	2	Q1	UCLA scale
		6	4	Median	
		6	6	Q3	
0.001	−3.193	3	1	Q1	Grimby scale
		4	3	Median	
		6	3	Q3	
0.114	−1.576	5	0	Q1	VAS Activity Scale
		6	4	Median	
		8	8	Q3	

Z—standardized value of the Mann–Whitney test; *p*-value for the Mann–Whitney U test; Q1, Q3—1st and 3rd quartile.

**Table 3:** Detailed functional assessment of patients before and after surgery—plate group.

*p* Value	Z	After Treatment	Before Treatment		Analyzed Variable
Plate Group					
0.001	3.123	2	2	Q1	UCLA scale
		3	4	Median	
		5	6	Q3	
0.001	3.196	2	3	Q1	Grimby scale
		2	3	Median	
		4	6	Q3	
0.001	3.649	3	1	Q1	VAS Activity Scale
		3	4	Median	
		6	8	Q3	

Z—standardized value of the Mann–Whitney test; *p*-value for the Mann–Whitney U test; Q1, Q3—1st and 3rd quartile.

**Table 4:** Selected functional parameters of patients with plate stabilization and patients using the Ilizarov method—functional parameters.

*p*	Z	Ilizarov Group	Plate Group		Analyzed Variable
0.854	3.372	2	2	Q1	UCLA scale before treatment
		4	4	Median	
		6	6	Q3	
0.817	3.242	1	3	Q1	Grimby scale before treatment
		3	3	Median	
		3	6	Q3	
0.778	2.965	0	1	Q1	VAS Activity Scale before treatment
		4	4	Median	
		8	8	Q3	
0.754	0.312	5	4	Q1	UCLA scale after treatment
		6	6	Median	
		6	7	Q3	
0.001	3.173	3	2	Q1	Grimby after treatment
		4	3	Median	
		6	4	Q3	
0.03	2.167	5	3	Q1	VAS Activity Scale after treatment
		6	5	Median	
		8	6	Q3	

Z—standardized value of the Mann-Whitney test; *p*-value for the Mann Whitney U test or Pearson ch2; Q1, Q3—1st and 3rd quartile.

**Table 5:** Selected functional parameters of patients with plate stabilization and patients using the Ilizarov method—parameters—clinical parameters—operated limb.

*p*	Z	Ilizarov Group	Plate Group		Analyzed Variable
0.155	1.422	10	9	Q1	Dorsiflexion OL [degree]
		18	13	Median	
		22	17	Q3	
<0.001	−4.404	10	30	Q1	Plantar flexion OL [degree]
		15	37	Median	
		25	44	Q3	
<0.001	−3.555	5	12	Q1	adduction OL [degree]
		10	15	Median	
		10	20	Q3	
0.002	−3.052	8	13	Q1	abduction OL [degree]
		9	17	Median	
		10	23	Q3	

OL—operated limb; Z—standardized value of the Mann-Whitney test; *p*-value for the Mann Whitney U test or Pearson ch2; Q1, Q3—1st and 3rd quartile.

**Table 6:** Selected functional parameters of patients with plate stabilization and patients using the Ilizarov method—parameters—clinical parameters—non-operated limb.

*p*	Z	Ilizarov Group	Plate Group		Analyzed Variable
<0.001	4.751	35	13	Q1	Dorsiflexion NOL [degree]
		40	16	Median	
		40	21	Q3	
<0.001	−3.399	25	41	Q1	Plantar flexion NOL [degree]
		30	47	Median	
		35	53	Q3	
0.016	−2.393	10	14	Q1	adduction NOL [degree]
		14	17	Median	
		15	24	Q3	
0.0125	−2.497	8	13	Q1	abduction NOL [degree]
		10	18	Median	
		17	23	Q3	

NOL—non-operated limb; Z—standardized value of the Mann-Whitney test; *p*-value for the Mann Whitney U test or Pearson ch2; Q1, Q3—1st and 3rd quartile.

**Table 7:** Detailed range of motion of patients in Ilizarov group.

*p*	Z	NOL	OL		Analyzed Variable
Ilizarov Group					
<0.001	4.396	35	10	Q1	Dorsiflexion [degree]
		40	18	Median	
		40	22	Q3	
<0.001	3.567	25	10	Q1	plantar flexion [degree]
		30	15	Median	
		35	25	Q3	
<0.001	2.758	10	5	Q1	adduction [degree]
		14	10	Median	
		15	10	Q3	
0.184	1.327	8	8	Q1	abduction [degree]
		10	9	Median	
		17	10	Q3	

OL—operated limb, NOL—non-operated limb; Z—standardized value of the Mann-Whitney test; *p*-value for the Mann Whitney U test; Q1, Q3—1st and 3rd quartile.

**Table 8:** Detailed range of motion of patients in Plate group.

*p*	Z	NOL	OL		Analyzed Variable
Plate Group					
0.132	1.503	13	9	Q1	Dorsiflexion [degree]
		16	13	Median	
		21	17	Q3	
0.048	1.97	41	30	Q1	plantar flexion [degree]
		47	37	Median	
		53	44	Q3	
0.082	1.737	14	12	Q1	adduction [degree]
		17	15	Median	
		24	20	Q3	
0.704	0.379	13	13	Q1	abduction [degree]
		18	17	Median	
		23	23	Q3	

OL—operated limb, NOL—non-operated limb; Z—standardized value of the Mann-Whitney test; *p*-value for the Mann Whitney U test; Q1, Q3—1st and 3rd quartile.

## Data Availability

The data presented in this study are available on request from the corresponding author.
